# Comparison of lentiviral vector titration methods

**DOI:** 10.1186/1472-6750-6-34

**Published:** 2006-07-12

**Authors:** Martine Geraerts, Sofie Willems, Veerle Baekelandt, Zeger Debyser, Rik Gijsbers

**Affiliations:** 1 Laboratory for Molecular Virology and Gene Therapy, K.U.Leuven and IRC KULAK, Flanders, Belgium; 2 Laboratory for Neurobiology and Gene Therapy, K.U.Leuven, Flanders, Belgium

## Abstract

**Background:**

Lentiviral vectors are efficient vehicles for stable gene transfer in dividing and non-dividing cells. Several improvements in vector design to increase biosafety and transgene expression, have led to the approval of these vectors for use in clinical studies. Methods are required to analyze the quality of lentiviral vector production, the efficiency of gene transfer and the extent of therapeutic gene expression.

**Results:**

We compared lentiviral vector titration methods that measure pg p24/ml, RNA equivalents/ml, transducing units (TU/ml) or mRNA equivalents. The amount of genomic RNA in vector particles proves to be reliable to assess the production quality of vectors encoding non-fluorescent proteins. However, the RNA and p24 titers of concentrated vectors are rather poor in predicting transduction efficiency, due to the high variability of vector production based on transient transfection. Moreover, we demonstrate that transgenic mRNA levels correlate well with TU and can be used for functional titration of non-fluorescent transgenes.

**Conclusion:**

The different titration methods have specific advantages and disadvantages. Depending on the experimental set-up one titration method should be preferred over the others.

## Background

In our laboratory we routinely produce and apply vectors derived from the human immunodeficiency virus type 1 (HIV-1). Since lentiviral vectors (LV) integrate stably into the host-cell genome of non-dividing cells such as neurons and in haematopoietic stem cells [1-3], they offer great potential for gene therapeutic applications [4]. For biosafety reasons, the HIV-1 genome has been modified and *cis *and *trans*-acting viral sequences have been segregated over 3 to 4 different plasmids [5,6]. Indeed, viral structural and functional proteins can be provided in *trans *and are encoded by 1 or 2 packaging plasmids while the envelope plasmid encodes the glycoprotein of the vesicular stomatitis virus envelope (VSV-G) and a transfer plasmid encodes the transgene of interest flanked by all *cis*-acting viral sequences necessary for packaging of the RNA genome (reviewed by [7]). Production of lentiviral vectors is routinely achieved by transient transfection of human embryonic kidney (293T) cells using high concentrations of the different plasmids, implicating the presence of residual plasmid DNA in the vector preparation, even after concentration. Transduction by lentiviral vectors matches a single-round infection and results in long-term integration into the genome of both dividing and non-dividing cells, forever linking the fate of the provirus with that of the target cell. VSV-G pseudotyping of the lentiviral vector particles not only broadens the tissue tropism of the vector, but also stabilizes the particles allowing concentration to high titers by ultracentrifugation [8]. Since the initial development of the lentiviral vector system [2,5,6] the transfer plasmid was gradually optimized in order to improve biosafety as well as to increase transduction efficiency. The self-inactivating (SIN) deletion in the 3' LTR [9] limits vector rescue and reduces the likelihood of promoter activation after integration. The woodchuck hepatitis virus posttranscriptional regulatory element (WPRE) [10] stabilizes the transgene mRNA and the insertion of the central polypurine tract/central termination site (cPPT/CTS) sequence stimulates nuclear import [11].

Approval of lentiviral vectors for cell-marking and therapeutic studies in humans requires in-depth characterization of vector titers and expression profiles of therapeutic genes. Ample methods to evaluate lentiviral vector titers have been described (reviewed by [12]). These methods can roughly be divided into functional and non-functional titration methods. The latter include p24 antigen ELISA, assessment of the reverse transcriptase activity and determination of the genomic RNA concentration in vector preparations by semi-quantitative northern blotting, dot blot analysis or RT-qPCR. Generally these techniques overestimate the functional vector titer and suffer from following disadvantages: the p24 protein pool that is quantified includes a variable amount of free p24 and p24 that originates from non-functional vector particles. Similarly, RNA titers will also assess defective particles, whereas the RT-assay merely demonstrates RT activity. A more accurate, functional titer is determined by transduction of cells following limiting dilution of vector and subsequent evaluation of reporter protein activity, (e.g. beta-galactosidase positive cells) or by assessment of the number of colony forming units following antibiotic selection. The most widespread and straightforward technique to quantify functional vector titers employs eGFP fluorescence and fluorescence-activated cell sorting (FACS). However, FACS analysis of transgene expression is restricted to fluorescent reporter proteins and cannot discriminate cells with single or multiple integrations. In strict sense, the definition of a functional vector titer is the number of vector particles required to infect a cell, present in a volume. In this regard, the best measurement of the number of functional particles can be accomplished by determination of the number of integrated proviral DNA copies per cell by qPCR [13-15]. However, due to insertion in regions with different chromatin packing, the integrated proviral DNA results in varying transgene expression levels. To overcome this drawback, Lizeé et al. [15] described a RT-qPCR method to quantify lentiviral mRNA copies following stable transduction in cell culture. Ultimately, the method of choice will depend on the experimental set-up. Basic research and possible clinical applications are in need of a universal, functional titration method for any transgene-of-interest, for example by qPCR. When analysing different internal promoters driving transgene expression quantification of the number of integrated proviral DNA copies following titration on a reference cell line is recommended. On the other hand, to compare different lentiviral vector backbones comprising additional *cis*-acting elements, a non-functional titration method is preferred to normalize the number of vector particles before assessing transduction efficiency.

In this study, we developed a quantitative RT-PCR assay, for quantification of both genomic lentiviral RNA after production and of transgene transcripts following transduction. We opted for a one-step RT-qPCR to reduce both sample handling time and variability. In addition, in contrast with the published methods, samples were amplified alongside a RNA standard to correct for low reverse transcriptase efficiency. The reliability of the different titration methods (RT-qPCR, ELISA and FACS) was evaluated and the methods were subsequently applied to assess vector production quantitatively and qualitatively. Next, we analysed the correlation between transgene expression as measured by FACS analysis and RT-qPCR. Although several groups have reported on the use of TU/ml or pg p24/ml to normalize vector transduction experiments [16,17], a careful side-by-side analysis was hitherto absent. Here, we normalized vectors for RNA and p24 values prior to transduction and evaluated the transgene expression to determine the best titration method to normalize lentiviral vectors.

## Results and discussion

### Validation of a one-step RT-qPCR to determine lentiviral RNA content in concentrated vector preparations

**Figure 1:**
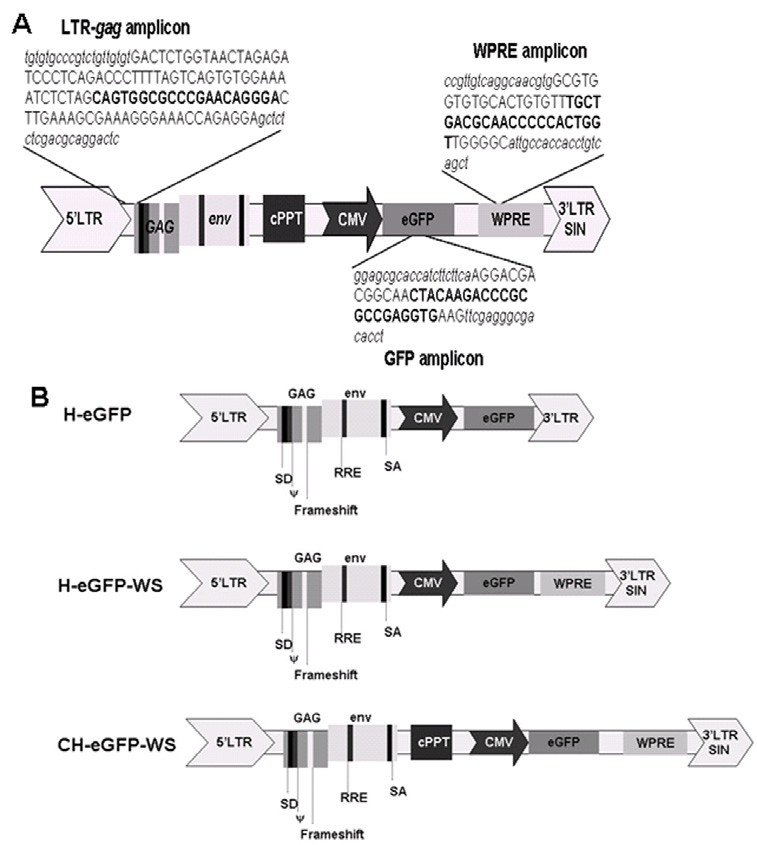
**Overview of primer sets and lentiviral vector constructs**. (A) Schematic representation of CH-eGFP-WS lentiviral vector with the corresponding amplicons of the different primer sets (LTR-*gag*, GFP and WPRE). Primer sequences are represented in small caps and probe sequences are in bold. (B) Schematic representation of different lentiviral vector constructs. The construct was optimized to increase transduction efficiency (cPPT and WPRE) and biosafety (SIN) as described before [9, 10, 23].

A one-step real-time RT-qPCR for quantification of lentiviral vector RNA in concentrated vector preparations was established. One-step RT-qPCR combines the reverse transcriptase reaction and the amplification in a single tube, reducing sample handling time and variability. Due to the exponential nature of PCR amplification, a highly specific and quantitative measurement in the linear range of amplification can be performed with a TaqMan Probe, labeled with a reporter fluorophore and a quencher at the 5' and 3' end, respectively. Primers and probe are directed against the U5 region of the 5' LTR and the 5' end of the *gag *gene, sequences that are present in all HXB2-derived lentiviral vector constructs [18] (Figure 1A). A linear relation between the copy number and the fluorescent signal intensity was observed over 6 logs (5.45 × 10^3 ^to 5.45 × 10^8 ^RNA equivalents/reaction with a slope = -3.2) (data not shown). Although quantification of lentiviral vectors by real-time PCR has been described before [14], we are the first to use an RNA standard. The use of an RNA standard takes into account the limiting amount of RNA that is actually reverse-transcribed into cDNA. Indeed, a DNA standard, as used in a two-step RT-qPCR, possibly underestimates the RNA copy number. Because lentiviral vectors are produced by triple transient transfection of 293T cells, plasmid DNA is present in the concentrated vector requiring a DNase treatment of each lentiviral vector sample prior to RT-qPCR (results not shown). Alternatively, plasmid DNA contamination could be overcome by the design of stable producer cell lines [19-21]. Next, the reproducibility of RNA extraction and RT-qPCR were validated by comparing the RT-qPCR results of a CH-eGFP-WS lentiviral vector subjected to three independent RNA extractions (Table 1). Subsequently, each sample was run in triplicate in the RT-qPCR. The coefficient of variation (CV) was 6 ± 4 % between triplicate samples of the same RNA extraction and 38 ± 22 % for three independent RNA extractions (data not shown).

Next, three different lentiviral vectors (H-eGFP, H-eGFP-WS and CH-eGFP-WS) were produced in parallel (Figure 1B). H-eGFP-WS contains the WPRE, known to affect mRNA stability [10] while the cPPT/CTS sequence in CH-eGFP-WS improves the transduction efficiency [22,23]. For each vector the RNA equivalents, transducing units (TU/ml) and p24 concentrations were determined to compare the different titration methods. Obviously, a clear difference between the lentiviral vectors was only evidenced by measuring the transducing titer (TU/ml), whereas the RNA and p24 concentration were similar for all vector backbones, pointing out that the packaging efficiency was comparable for the different constructs. In addition, although each functional vector particle (1 transducing unit) carries two RNA copies implying a theoretical ratio of 0.5, in reality the TU/RNA ratio ranged between 0.0009 and 0.0832 (Table 1). The TU/pg value ranged between 11 and 351. Both TU/pg and TU/RNA estimate the specific activity and correlate well with improved lentiviral vector backbone design. Table 1 shows a 6 and 8-fold increase in specific activity, when comparing the H-eGFP with H-eGFP-WS vector and a 31 and 68-fold increase when comparing the H-eGFP with the CH-eGFP-WS vector for TU/pg and TU/RNA respectively. Although the specific activities correlate well with the vector backbone, the differences between TU/pg and TU/RNA demonstrate that this is not an absolute value. Indeed, variations in TU, p24 and RNA titer may also be attributed to the inherent variability of transient transfection used for vector production, which is also dependent on the number of cells plated or the state of the producer cells. The TU/pg and TU/RNA values thus give an indication of the quality of the vector production but are subjected to the variable amounts of p24 and RNA produced by the cells.

It has been shown before that RNA values overestimate functional eGFP titers (TU/ml) by 200- to 10,000-fold [13-15,24]. In our hands, using a RNA standard, we detected an approximately 10- to 1000-fold difference between the eGFP and RNA titers depending on the vector backbone. The discrepancy between the RNA and TU titer between several groups may be dependent on the vector backbone or other factors. First, the possibility exists that incomplete, defective genomes are integrated in the vector particles [25]. Second, during transduction, part of the functional vector particles may stay in the cell culture medium and it has been shown that changes in inoculum volume and transduction time all influence transducing titers [17]. Third, for lentiviral vectors it was shown previously by two independent groups that only ~10% to ~18% of the initial reverse transcribed genomes actually integrate in the host-cell DNA of 293T cells after transduction, probably due to degradation in the cytoplasm [18,26]. Fourth, not all integrated proviral genomes may result in detectable transgene expression. Several groups, except for one [14], demonstrated that the proviral-based qPCR overestimates eGFP titers varying from 6-to 60-fold [13,15,27], probably due to integration in DNA regions with reduced transcriptional activity.

### Quantification of genomic lentiviral vector RNA using different primer sets

**Figure 2:**
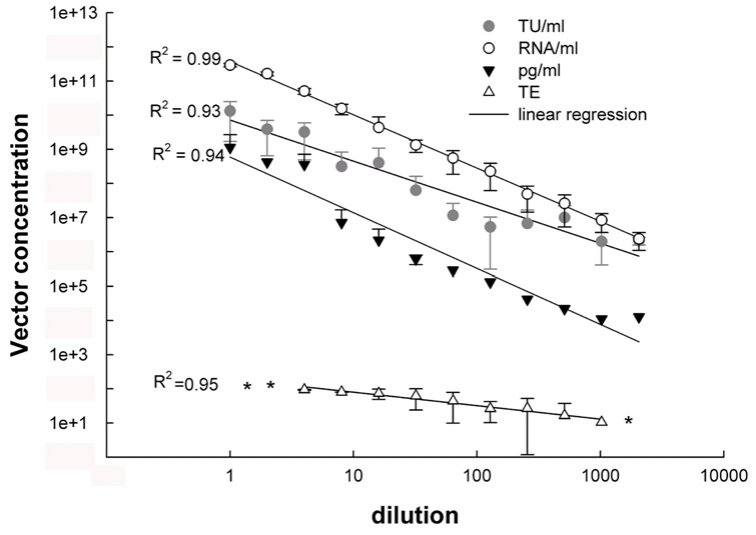
**Comparison of lentiviral vector titration methods**. CH-eGFP-WS vector was serially diluted (1/2) and subjected to RT-qPCR, ELISA and FACS analysis after transduction of 293T cells, to determine the linearity of the different titration methods. The correlation coefficients are representative for 3 independent experiments. The asterix (*) represents values that were not included in the linear regression.

The amplicon of the primer set that is used to quantify the lentiviral vector RNA is located in the 5'LTR of the RNA genome. Hence, lentiviral vector RNA containing a packaging signal but truncated at the 3' end can still be incorporated into vector particles, thereby affecting both RNA titers and p24 values, but eventually resulting in non-functional vectors. Therefore, the *LTR*-*gag *primer/probe set was compared to a primer/probe set directed against the *eGFP *transgene and a *WPRE *primer/probe set (Figure 2A) on the same plasmid DNA standard to reduce variation between different standards. As shown in Table 2, all primer sets were equally efficient in amplifying the pCH-eGFP-WS plasmid DNA. Next, we quantified lentiviral vector RNA titers (in triplicate) by one-step RT-qPCR for two independent CH-eGFP-WS vector preparations comparing the three primer sets. Cycle threshold values (C_t_, *i.e*. the cycle number at which a significant increase in fluorescence above base-line signal is detected) did not differ significantly between the WPRE, LTR-gag or eGFP primer sets. To control for contaminating mRNA transcripts from producer cells that may be concentrated together with the vector particles, we performed a transient transfection without packaging plasmid. Since expression from our transfer plasmid is Tat-dependent, omission of the packaging plasmid, resulted in a 1000-fold (≥ 9 Ct) reduction in vector titers as quantified with the LTR primers. Contamination with eGFP- mRNA, transcribed from the internal CMV promoter, was verified in the same experiment but using eGFP primers. A 100-fold (≥ 6 Ct) reduction in eGFP-mRNA was detected indicating that eGFP-mRNA contaminates the vector preparations to a slightly higher extent. Still, the great majority of the amplified cDNA is derived from full-length RNA constructs that are incorporated in the viral particles. Therefore, the discrepancy between RNA and functional titer (Table 1) is not due to the presence of incomplete genomic RNA.

### Comparison of lentiviral vector titration methods

Most frequently used titration methods for lentiviral vectors measure the p24 antigen concentration (pg p24/ml) by ELISA or the number of transducing units (TU/ml) by FACS analysis after limiting dilution in cell culture. Whereas the p24 concentration measures both functional and non-functional vector particles, the TU strictly measures functional vector particles that result in the expression of a fluorescent reporter protein. To compare the linearity, reproducibility and variability of the different methods, a CH-eGFP-WS lentiviral vector was serially diluted (12 steps of 1/2 dilution) and subjected to RNA extraction, p24 ELISA and transduction in cell culture. All titration methods correlated well with the initial dilution series: r^2 ^= 0.99 for RNA/ml after RT-qPCR, r^2 ^= 0.93 for TU/ml after FACS and r^2 ^= 0.94 for p24/ml after ELISA (Figure 2). When determining transduction titers after limiting dilution, one uses only dilutions at MOI<<1 resulting in low percentage of transduced cells, to minimize the risk for multiple integrations. Titration depends on the total volume that is covering the cells, the time of incubation with the particular vector dilution and the cell type used. Prolonged incubation or delivery at the same MOI in only half of the volume will affect titers significantly. Nevertheless, using standardized conditions, these methods allow a good estimation of vector quality and titers. As a control, we checked the correlation between vector dilution and transduction efficiency (TE, i.e. the percentage of transduced cells) as measured by FACS. The most concentrated vector dilutions resulted in near 100% transduction, whereas the most diluted samples resulted in TEs near zero (* in Figure 2) and were omitted from the linear regression. Hence, the TE correlated only over 9 dilutions with r^2 ^= 0.95.

Moreover, to estimate the variability within each test, we calculated the coefficients of variation (CV) for the different methods. The CV for the RNA/ml, the TU/ml and the pg p24/ml were respectively 39%, 78% and 103% on average. In conclusion, the RNA and p24 concentration as well as the functional titer are reliable parameters to assess the order of magnitude of vector titers. However, absolute numbers differ between samples as shown by the CV. The highest CV was obtained for the p24 ELISA. Other disadvantages of the p24 measurement are the restricted linear range (13–200 pg/ml) and the accompanying extensive dilution of the concentrated vector sample that is required and affects reproducibility. The high CV for the TU/ml may be due to variations in the cell number upon transduction or random integration in the genome, resulting in differences in transgene expression level.

### Analysis of gene expression after normalization for p24 or vector RNA concentration

To evaluate vector optimization (promoter choice, insertion of enhancer elements) it is important to normalize vector preparations prior to transduction. Functional titration methods such as FACS analysis after limiting dilution cannot be used in this case, since this titer is dependent on the specific backbone. Here we compared the use of p24 and vector RNA concentration for normalization of CH-eGFP-WS vectors prior to transduction. Separate productions of CH-eGFP-WS-derived lentiviral vector were prepared and RNA equivalents and p24 concentration determined within a single test run to minimize inter-assay variation. The percentage of eGFP-positive cells was determined by FACS 3 days later (Table 3). A high variation was observed between the different productions amounting to a CV of around 50% for both techniques. The variability is probably a combination of variation due to the triple transient transfection procedure, which results in variable protein expression and viral genome production for separate productions, and variation of the technique itself (see before). High variations in p24 values were already described by Logan and colleagues [16] who measured p24 values ranging from 67.4 ng/ml to 583.7 ng/ml even for vectors produced in parallel. Moreover, the relative transduction values for the productions differ for both RNA and p24 measurements, indicating a poor correlation between both parameters and reflecting the fact these are dependent on transfection of transfer and packaging plasmid, respectively. Both p24 and RNA concentrations can be used for comparison of vector performance if minimizing intrinsic variability of the test by single run analysis and vector productions in parallel. In conclusion, both the RNA titer and p24 values are poor in predicting gene transfer efficiency. Still, they can be used for normalization of vector preparations.

### Evaluation of the reliability of the titration methods to assess lentiviral vector production quality and kinetics

**Figure 3:**
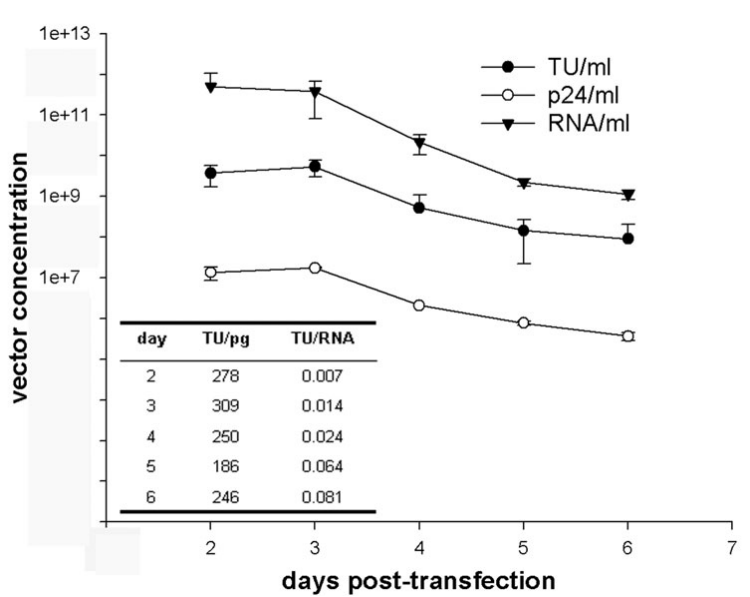
**Kinetics of lentiviral vector production**. The CH-eGFP-WS-derived lentiviral vector was produced in two CF2 modules by triple transient transfection in serum-free medium. Starting at 2 days post-transfection, cell-culture medium was harvested once a day for five consecutive days and concentrated by low-speed centrifugation. The p24 content (pg p24/ml) of vector preparations was determined by ELISA, the transducing titers (TU/ml) by FACS analysis and the RNA equivalents (RNA/ml) by one-step RT-qPCR with LTR primers. Data represent the mean value ± standard deviation and represent 3 independent experiments. A decrease in vector titer starting at 3 days post-transfection was evidenced by all methods. The specific activities (TU/pg and TU/RNA) are depicted in the inset.

In our vector core lentiviral vectors are produced on a weekly basis. Cell supernatant containing lentiviral vectors is routinely harvested in serum-free medium at 2 and 3 days after triple transient transfection [8]. To analyze the kinetics of lentiviral vector productions in further detail and to investigate the possibility of harvesting for a longer time period, a CH-eGFP-WS-derived vector was produced in two 2-layer cell-factories, as described earlier [8]. The supernatant was harvested once daily for five consecutive days (day 2 till day 6 post-transfection) and subsequently concentrated by low-speed centrifugation (5 hrs, 26,000 g). Figure 3 displays the kinetics of the different parameters during days of production. From day 3 onwards, vector titers start to decline, as evidenced by the three titration methods. Notwithstanding the drop in vector titers, there is still a significant amount of high-quality vector produced at day 4 and 5. The specific activity in TU/RNA increases at each harvesting day, whereas the specific activity in TU/pg displays comparable values each day (Figure 3). These results indicate that at later time points, vector with the highest specific activity (TU/RNA) is produced. One can envision that transient transfection will not be optimal at later days of production and expression will be gradually lost upon further division of the producer cells, resulting in a 10- to 100-fold decline in vector concentrations (TU/ml, RNA/ml and pg p24/ml). In addition, small loss of producer cells upon medium replacement will further result in decreased titers in time. Based on these results and for practical reasons, we decided to harvest our state-of-the-art vectors at day 2 and at day 3 post-transfection since for most experiments high-titer vector is needed.

Besides quantifying lentiviral vector concentration, the optimized RT-qPCR also provides us with an alternative method to evaluate vector quality after production. Indeed, most transfer plasmids encode non-fluorescent transgenes, leaving the p24 concentration as the only quality control. To assess the reliability of the different titration methods to analyse vector production quality, a CH-eGFP-WS vector was produced in 2-layer cell factories by triple transient transfection of the envelope, the packaging and the transfer plasmid. In parallel with the standard production, three additional productions were performed replacing either one of the three plasmids with a control plasmid in order to maintain a constant transfection efficiency. Titers were determined (pg p24/ml, RNA/ml and TU/ml) following vector concentration by low-speed centrifugation (Table 4). Whereas the standard vector production generated fully functional vector particles as demonstrated by the functional titer (TU/ml), omission of any of the other plasmids during production resulted in no detectable functional titers. As expected, omitting the transfer plasmid (encoding the viral vector genome) during production resulted in the production of empty, non-functional vector particles, as reflected by the absence of both a functional titer (TU/ml) and an RNA titer. Surprisingly, the p24 concentration was not significantly different from the standard vector. Indeed, the mere presence of the packaging plasmid resulted in normal p24 values in all production protocols. Exclusion of the envelope plasmid encoding VSV-G during the vector production resulted in p24 and RNA titers comparable to those of a normal vector production, whereas the functional titer remained below detection limit (TU/ml), inferring the generation of vector particles defective in cellular uptake. Furthermore, this result indicates that transduction by our lentiviral vector is not skewed by pseudo-transduction with free eGFP, DNA or RNA. Obviously, omission of the packaging plasmid resulted in the absence of p24 antigen and the generation of non-functional particles (no TU/ml). Although the RNA titer obtained was reduced by three logs, RNA transcripts apparently contaminate the vector preparation to a limited extent. This is in accordance with results from Ikeda *et al *[24], who also demonstrated that without Gag-Pol the RNA secretion represented only 0.1 % of the packaged RNA. In addition, this result confirms the conclusions drawn from table 2, i.e. mainly full-length RNA transcripts are incorporated in lentiviral vector constructs.

Our results clearly demonstrate that the p24 concentration is the least reliable for the evaluation of functional vector particles after vector production. Nevertheless, it is a fast method that can be used as quality control for routine vector production. Logan and colleagues [16] described how manipulation of the amounts of transfer, packaging or envelope plasmids did not alter the specific activity (TU/pg p24) but rather influenced the vector concentration in the supernatant (TU/ml and pg p24/ml). However, if non-fluorescent transgenes are encoded, a functional titer cannot be determined, and one has to rely on the RNA concentration indicating the presence of genomic RNA. How this RNA titer relates to the functional titer is dependent on the vector construct (see Table 1), the transgene and the promoter, and requires further analysis of the integrated proviral genome by qPCR or of the transgene expression level by RT-qPCR, Western blotting or immunocytochemistry after transduction.

### Evaluation of transgene expression by RT-qPCR

**Figure 4:**
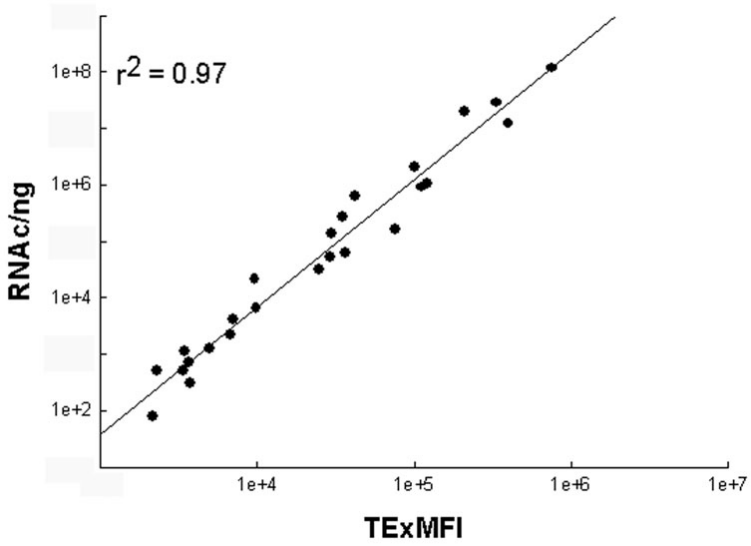
**Correlation of eGFP fluorescence and mRNA transgene expression levels**. Four independently produced preparations of CH-eGFP-WS-derived lentiviral vector were serially diluted (1/10) prior to transduction of 293T cells. 6 days later, cells were harvested for eGFP analysis by FACS or RNA extraction and subsequent RT-qPCR with primers and probe directed against WPRE to measure transgene expression and against RNAse P to normalize for total RNA content. Total eGFP expression was measured by multiplying the percentage of transduced cells (TE) with the mean fluorescence intensity (MFI). The transgene mRNA level is given as the number of mRNA copies normalized to the total RNA content (RNAc/ng). These values correlate strongly with each other (r^2 ^= 0.97).

Eventually, future clinical applications of gene transfer vectors will necessitate accurate determination of expression levels of any therapeutic transgene. Lizee and collegues [15] successfully applied a quantitative and generic two-step RT-qPCR method (with a DNA standard) to determine mRNA expression levels in transduced cells with a primer-probe set located in the WPRE, present in the 3' UTR of the transgenic mRNA. In contrast, we optimized a one-step RT-qPCR also with a WPRE primer-probe set but with a RNA standard to quantify expression levels (Figure 1A). To assess reproducibility, a CH-eGFP-WS lentiviral vector was serially diluted (1/10) on 293T cells. Transgene expression (eGFP) was measured both by FACS and by RT-qPCR. A primer-probe set designed for human RNase P, a housekeeping gene, was added to each sample to allow normalization to the total RNA content. Again, an RNA standard (5 × 10^3 ^to 5 × 10^8 ^RNA equivalents/reaction with a slope = -3.2) was taken along with samples and no amplification controls. Subsequently, relative values were calculated and presented as the number of RNA copies per ng total RNA (mRNAc/ng). The over-all transduction efficiency was calculated by multiplying the percentage of eGFP-positive cells (TE) and the mean fluorescence intensity (MFI). As demonstrated in Figure 4, lentiviral vector transgene expression (as measured by FACS) and transgene RNA expression (as measured by RT-qPCR) strongly correlate (correlation coefficient r^2 ^= 0.97), indicating that this method can be used to measure non-fluorescent transgene expression. Differences in MFI are observed and are related to the amount of eGFP expressed in the cells, which may vary depending on the integration site or the integrated copy number and depend on the internal promoter and the cell type. Differences in mRNA expression levels are also depending on these factors, as shown by the correlation between TE and mRNAc/ng (r^2 ^= 0.7). Hence, this method is an alternative for FACS analysis of fluorescent genes but does not discriminate between multiple integrations. Several DNA-based quantitative PCRs for integrated proviral genomes are described but are known to overestimate eGFP titers by 6- to 60-fold [13,15,27], since not all integrated vectors contribute to active gene expression. Therefore, the RNA transgene expression level correlates best with the actual protein expression level and can replace FACS analysis for non-fluorescent transgenes, although this method is more laborious and more expensive. Moreover, a more than 50-fold difference in vector titer (TU/ml) was reported by changing the conditions of the transduction process, such as inoculum volume, the type and number of target cells and the length of vector exposure to target cells [17]. Therefore standardization of the exact procedure of titration remains of uttermost importance.

## Conclusion

In this paper, commonly used methods for titration of lentiviral vectors were evaluated and may serve as a guide for newcomers in the field. For basic studies and eventually clinical trials, it is imperative that the performance characteristics and the variability inherent with these titration methods are known.

Due to the transient nature of a lentiviral vector production, variations in p24, TU and RNA levels inherent to the production procedure were demonstrated by ELISA, FACS analysis and RT-qPCR, respectively. These methods can be used to measure vector titers, although absolute numbers may vary even within the same run. Nor RNA nor p24 titers, can predict a functional titer, since the functional titer is dependent on the vector construct and the cell type used for transduction. The TU/RNA and TU/pg ratios reflect the specific activity of a lentiviral vector construct and were demonstrated to correlate well with the vector backbone. Normalization of vector productions based on RNA or p24 values are hampered by variability due to the transient transfection and the titration method and should be taken into consideration. Moreover, to assess the quality of lentiviral vector productions encoding non-fluorescent genes, the RNA titer is the most reliable, since p24 titers were not affected by omission of the transfer plasmid. We also demonstrated that the quantitative analysis of transgene mRNA levels correlates very well with the eGFP fluorescence as measured by FACS and hence can be used as an alternative for titration of vectors encoding non-fluorescent transgenes or determination of the transgene expression levels in transduced cells.

## Methods

### Cell lines and lentiviral vector transduction

293T cells were maintained in Dulbecco's modified Eagle's medium (DMEM; Gibco BRL, Merelbeke, Belgium) supplemented with 10% heat-inactivated foetal calf serum (FCS, Harlan Sera-Lab Ltd., International Medical, Brussels, Belgium) and 100 U/mL Penicillin and 100 μg/mL Streptomycin (Gibco BRL). Chinese hamster ovary cells, CHO-K1 were maintained in Ham's F12K medium (NutMix F12, Gibco BRL) supplemented with 5% heat-inactivated FCS and 20 μg/ml gentamicin (Gibco BRL). Both cell lines were cultivated at 37°C in a humidified atmosphere containing 5% CO_2_. Transductions of CHO-K1 or 293T cells with HIV-1 based lentiviral vectors were carried out in 96-well plates with 10-fold serial dilutions of lentiviral vector preparations. Vector was added to the cells in their corresponding medium supplemented with 1% FCS. After 4 hours of incubation, the medium was refreshed. Three days later, cells were removed and fixed in 2% paraformaldehyde prior to determination of titers (TU/ml) by limiting dilution and FACS.

The coefficient of variation (relative standard deviation) was determined for the different titration methods, which is a measure for the biological and experimental variation and is calculated as follows: stdev/mean × 100.

### Lentiviral vector production

HIV-1-derived lentiviral vector particles, pseudotyped with the VSV-G envelope, were produced by transfecting 293T cells with a second generation packaging plasmid lacking *vif, vpr, vpu *and *nef *genes (pCMVΔR8.91), a plasmid encoding the envelope of VSV (pMDG) and a pHR'-derived transfer plasmid coding for eGFP as a reporter gene. Lentiviral vectors were produced as described earlier [8]. Lentiviral vector stocks were normalized based on p24 antigen content (HIV-1 p24 ELISA kit; PerkinElmer, Milano, Italy). Transduction titers for vectors encoding eGFP were determined by FACS analysis after limiting dilution on CHO-K1 cells.

### One-step real-time RT-qPCR

RNA content of lentiviral vectors was quantified with a one-step RT-qPCR that allows reverse transcription (RT) and amplification to take place in the same reaction tube. After RNA extractions of concentrated vector preps with the RNAqueous^®^-Micro Kit (Ambion, Cambridgeshire, United Kingdom) a DNase treatment (RNAqueous-Micro Kit) was carried out to eliminate residual plasmid DNA from the vector production according to the manufacture's protocol. RNA was amplified using the TaqMan^® ^One-Step RT-PCR master mix reagents kit (Applied Biosystems, Lennik, Belgium) with primers and probe located in the eGFP transgene, the LTR or the WPRE. Forward and reverse primers were developed with the Primer Express Software (Applied Biosystems) and are specific for eGFP: 5'- GGAGCGCACGATCTTCTTCA-3' and 5'-AGGGTGTCGCCCTCGAA-3'; for LTR 5'-TGTGTGCCCGTCTGTTGTGT-3' and 5'-GAGTCCTGCGTCGAGAGAGC-3' [18]; and for WPRE 5'-CCGTTGTCAGGCAACGTG-3' and 5'-AGCTGACAGGTGGTGGCAAT-3' [15]. Following TaqMan probes were used : for eGFP, 5'-FAM-CTACAAGACCCGCGCCGAGGTG-TAMRA-3'; for LTR, 5'-FAM-CAGTGGCGCCCGAACAGGGA-TAMRA-3' [18] and for WPRE, 5'-FAM-TGCTGACGCAACCCCCACTGGT-TAMRA-3' [15]. Amplicon sizes are for eGFP: 75 base pairs (bp), for LTR: 143 bp for WPRE: 85 bp. The kit contains the Multiscribe™ Reverse Transcriptase (MuLV) which carries out the RT-step (30 minutes at 48°C) and AmpliTaq Gold enzyme for amplification (40 cycli at 95°C for 15 seconds, followed by 1 minute at 60°C). Reactions were analyzed using the ABI Prism model 7700 sequence detection system (Applied Biosystems). During each run a 'no amplification control' (NAC) was included for each sample (i.e. sample without RT-enzyme) to detect residual DNA contaminants and this value was subtracted. To normalize the mRNA values of expressed transgenes to the total RNA content in the sample, additional primers and a VIC-TAMRA-labeled probe directed against the RNAse P housekeeping gene were included in the sample mixture (RNAse P control reagents kit, Applied Biosystems) and values were corrected for the total amount of RNA in the sample. (FAM: 6-carboxyfluorescein; TAMRA: 6-carboxytetramethylrhodamine)

### Generation of RNA standards by *in vitro *transcription

For quantification of the extracted vector, an RNA standard was prepared by *in vitro *transcription. Therefore, an LTR- (302 bp) or WPRE-fragment (640 bp) was cloned into pSPT19 or pBluescript, respectively. The plasmid was linearized by a restriction digest with an enzyme located downstream of the cloned fragment, followed by *in vitro *transcription according to the manufacturer's protocol (RNA labelling kit, Roche, Brussels, Belgium). The resulting RNA was quantified using a spectrophotometer and standard curves were generated by 1/10 serial dilutions. During each one-step RT-qPCR, the RNA standard was run together with the samples in duplicate and the NACs.

## Authors' contributions

MG developed the one-step RT-qPCRs, the RNA standards and drafted the manuscript. SW carried out the RT-qPCRs, the lentiviral vector productions and titrations by FACS. VB participated in the evaluation of the results. ZD and RG participated in the design of the study and helped to draft the manuscript. All authors have read and approved the final manuscript.

## Figures and Tables

**Table 1:** Evaluation of the different titration methods

	**RNA c/ml**	**TU/ml**	**pg p24/ml**	**TU/pg**	**TU/RNA**
*Cell factories*					
CH-eGFP-WS	2.68 ± 0.38 × 10^10^	2.23 ± 1.10 × 10^9^	9.8 ± 5.3 × 10^6^	228	0.0832
*Culture dishes*					
H-eGFP	5.63 ± 0.50 × 10^10^	5.05 ± 4.9 × 10^7^	4.67 ± 4.5 × 10^6^	11	0.0009
H-eGFP-WS	3.83 ± 2.25 × 10^10^	2.92 ± 2.5 × 10^8^	4.83 ± 4.70 × 10^6^	60	0.0076
CH-eGFP-WS	2.73 ± 1.59 × 10^10^	1.68 ± 1.3 × 10^9^	4.79 ± 3.45 × 10^6^	351	0.0615

The lentiviral vector CH-eGFP-WS was produced in cell factories and concentrated by centrifugation as described before [8]. Three independent RNA extractions were carried out on this vector and RNA equivalents were determined by RT-qPCR. Mean values ± standard deviation are shown. Next, three lentiviral vectors with different transfer plasmids, H-eGFP, H-eGFP-WS and CH-eGFP-WS, were produced in parallel in cell culture dishes. RNA equivalents (RNA/ml), transducing units (TU/ml) and p24 concentrations (pg p24/ml) were determined by RT-qPCR, titration and ELISA, respectively. The TU/pg and TU/RNA value indicate the specific activity of the vector constructs and correlate well with the vector backbones. The data represent the mean values ± standard deviation of three independent productions per lentiviral vector.

**Table 2:** Measurement of viral RNA in concentrated lentiviral vector preparations

	**C_t _value**		
	
**DNA Standard**	**LTR primers**	**eGFP primers**	**WPRE primers**
5.0 × 10^8^	15.51 ± 0.01	15.65 ± 0.04	15.45 ± 0.06
5.0 × 10^7^	17.57 ± 0.01	17.79 ± 0.10	17.51 ± 0.01
5.0 × 10^6^	18.97 ± 0.06	19.11 ± 0.08	20.11 ± 0.05
5.0 × 10^5^	22.06 ± 0.17	21.82 ± 0.07	22.59 ± 0.03
5.0 × 10^4^	25.91 ± 0.05	24.06 ± 0.06	26.13 ± 0.04

**RNA extracts**			
**CH-eGFP-WS**	22.87 ± 0.05	22.5 ± 0.02	22.04 ± 0.03
**CH-eGFP-WS**	16.15 ± 0.02	16.57 ± 0.03	16.43 ± 0.05

Primer/probe sets annealing to the front (LTR), the centre (GFP) or at the end (WPRE) of the genomic RNA of lentiviral vectors were used to determine to what extent full-length genomic vector RNA is incorporated into lentiviral vector particles. In one-step RT-qPCR assays with the different primer/probe sets comparable threshold cycles (C_t_) were detected when amplifying a dilution series of the pCH-eGFP-WS transfer plasmid (DNA standard) or RNA extracts of two representative CH-eGFP-WS vector preparations. Mean Ct values ± standard deviation for 3 amplifications of the same sample are shown.

**Table 3:** Evaluation of the transduction efficiency after normalization for RNA or p24

	**Equal p24**		**Equal RNA**	
**Vector production number**	**TE %**	**relative TE**	**TE%**	**relative TE**

11A	39.17	1.00	18.02	1.00
11B	4.67	0.12	4.2	0.23
116	29.6	0.76	21.86	1.21
126	11.19	0.29	10.64	0.59
78	31.18	0.80	9.15	0.51

median ± SD	30 ± 15		11 ± 7	

CH-eGFP-WS-derived lentiviral vectors were produced in separate productions indicated by a production number. RNA and p24 concentrations were determined in the same assays to reduce inter-assay variation. Before transduction of 293T cells, vectors were normalized for RNA or p24 concentration. Two days later, cells were harvested to determine the percentage of transduced cells by FACS (TE or transduction efficiency). TE values relative to the first production are presented as well. A high variation was observed after normalization for either RNA or p24.

**Table 4:** Reliability of the titration methods to assess lentiviral vector production quality

**packaging plasmid**	**envelope plasmid**	**transfer plasmid**	**RNA/ml**	**TU/ml**	**pg p24/ml**
+	+	+	6.18 ± 1.71 × 10^9^	1.07 ± 0.53 × 10^7^	4.10 ± 2.05 × 10^5^
+	+	-	below detection limit	below detection limit	2.10 ± 0.64 × 10^5^
+	-	+	2.36 ± 0.63 × 10^10^	below detection limit	1.27 ± 0.32 × 10^5^
-	+	+	8.04 ± 1.64 × 10^6^	below detection limit	below detection limit

Lentiviral vectors were produced in parallel in cell culture dishes by triple transient transfection with transfer, envelope and packaging plasmids. Omission of a plasmid is indicated. RNA equivalents (RNA/ml), transducing units (TU/ml) and p24 concentration (pg p24/ml) were determined after concentration of LV by low-speed centrifugation. For the CH-eGFP-WS vector, the titer was measured with all three methods. In the absence of the packaging plasmid, encoding for structural proteins, the RNA titer decreased 1000-fold while p24 and TU titers were below detection limit. Omission of the envelope plasmid during the vector production resulted in p24 and RNA titers comparable with those of a normal production albeit with a non-detectable functional titer. Vector production without transfer plasmid only yielded a positive p24 titer. Mean values ± standard deviation for 3 measurements of the same sample are shown.
